# Shrinkage of Prostate and Improved Quality of Life: Management of BPH Patients with *Croton membranaceus* Ethanolic Root Extract

**DOI:** 10.1155/2015/365205

**Published:** 2015-04-02

**Authors:** George Awuku Asare, Daniel Afriyie, Robert A. Ngala, Alfred A. Appiah, Yvonne Anang, Iddi Musah, Samuel Adjei, Kwabena Bamfo-Quaicoe, Derick Sule, Ben A. Gyan, Peter Arhin, Dominic A. Edoh

**Affiliations:** ^1^Department of Medical Laboratory Sciences, University of Ghana School of Biomedical and Allied Health Sciences, Korle Bu, Accra, Ghana; ^2^Ghana Police Hospital, Cantonment, Accra, Ghana; ^3^Department of Molecular Medicine, Kwame Nkrumah University of Science and Technology, Kumasi, Ghana; ^4^Center for Plant Medicine Research, Mampong, Akuapem, Ghana; ^5^Noguchi Memorial Institute for Medical Research, University of Ghana, Accra, Ghana; ^6^Department of Radiography, School of Biomedical and Allied Health Sciences, University of Ghana, Accra, Ghana; ^7^Traditional and Alternative Medicine Council, Ministry of Health, Accra, Ghana

## Abstract

Benign prostatic hyperplasia (BPH) is an enlargement of the prostate. The study aimed at validating the use of freeze-dried *Croton membranaceus* ethanolic root extract for BPH management. Thirty-three patients were observed before and after 3-month administration of 20 mg t.i.d orally. The International Prostate Symptom Score (IPSS), and the International Index of Erectile Function (IIEF) questionnaires were used. Total/free PSA (tPSA, fPSA), renal, liver function, lipid tests, and ultrasonographic imaging were performed. Thirty (30) patients (66 ± 11 years) completed the study. IPSS results showed 37% had severe, 40% moderate, and 23% mild symptoms before; 57% and 43% had moderate and mild symptoms, respectively, after treatment. IIED of patients' results showed 30% with severe, 40% moderate, 24% mild-moderate, 3% mild, and 3% no erectile dysfunction before treatment and 20% severe, 43% moderate, and 37% mild-moderate dysfunction, after treatment. Quality of life (QoL) improved (*P* = 0.001). Significant but non-pathological increases in total and indirect bilirubin as well as apolipoprotein A occurred. Mean tPSA reduced from 27.9 ± 19.0 to 16.2 ± 11.8 ng/mL (*P* = 0.002); fPSA from 6.1 ± 4.8 to 3.9 ± 2.9 ng/mL (*P* = 0.045); and prostate volume from 101.8 ± 41.3 to 54.5 ± 24.8 cm^3^  (*P* = 0.023). *C. membranaceus* shrinks the prostate and improves QoL.

## 1. Introduction

BPH is an enlargement of the prostate gland from progressive hyperplasia or abnormal growth of cells of glandular epithelial and stromal cells [1, 2]. Commonly experienced symptoms include inability to delay urination, incomplete emptying of the bladder, frequent urination during the day and night, weak urine stream, incontinence, and painful or bloody urination [3].

Globally, it has been documented that more than 80% of men by the age of 80 years will suffer from BPH [4]. Although BPH has a wide variety of treatment options from surgical to complementary alternative medicine (CAM), the high urologist to patient ratio, as well as socioeconomic problems that plaque developing economies, tend to drive the majority of the BPH patients towards dependency on CAM as the first line of medical relief. Indeed, CAM and the use of medicinal plants appear to be increasing worldwide. Furthermore, most men are reluctant to opt for surgical interventions for fear of losing potency and the perception of other adverse side effects. The use of 5*α*-reductase inhibitors and *α*-1 blockers for BPH management has side effects including erectile dysfunction (ED) [5, 6]. Against this background, subjects suffering from BPH appreciably resort to CAM, with as much as 90% of men in Austria and Germany depending on phytotherapy [7].

The role of phytotherapy in the health care delivery system is increasing. It is estimated that the global market for medicinal plants is worth over $250 billion [8]. Although the economic data does not exist in Ghana, medicinal plant pervoyeurs as well as their clients have increased. BPH patients in Ghana resort to the use of medicinal plants or a combination of medicinal plants and radiotherapy [9].

Traditional pharmacologic therapies although widely used must be assessed for a favorable risk-benefit ratio. One such plant that has been studied over three decades in Ghana is* Croton membranaceus* Mull. Arg. (Euphorbiaceae). This plant is mostly available in three west African countries, namely, Ghana, Nigeria, and Niger. In Ghana the plant is mostly found in the Krobo-Gyakiti forest reserve area in the Eastern Region. The root preparation of this medicinal plant has been dispensed to BPH patients at the Center for Plant Medicine Research (a WHO Center) for the past 30 years [10].

The root extract is said to contain an alkaloid, a coumarin, diterpenoids, and phytosterols [11–14]. Compounds isolated from the root extract include julocrotine, scopoletin, DL-threitol, crotomembranafuran, gomojoside H, larixol, *β*-sitosterol, *β*-sitosterol-3-*O*-glucoside, stigmasterol, and campesterol. Furthermore, N[N-(2-methylbutanoyl) glutaminoyl]-2-phenylethylamine has recently been isolated [15]. The crude ethanol extract of* C. membranaceus* and julocrotine possess 5-*α*-reductase inhibitory activity [11]. 5-*α*-reductase inhibitory activity is the underpinning mechanism for the application of finasteride for the treatment of BPH. BPH treatment with the aqueous root extract alongside finasteride demonstrating similar efficacy as finasteride has been reported in an animal model of BPH [16]. Furthermore, the antiproliferative activity of* C. membranaceus* on BPH-1 cells has been demonstrated [17]. Additionally, the cytotoxic activity of the methanolic extract of* C. membranaceus* roots has been shown against DLD-1 and MCF-7 cells. Finally, the aqueous root extract of the plant is nontoxic [18]. However, no published data exist on its effect on human BPH patients. This study aimed at observing the socioscientific markers of BPH patients opting for the use of the ethanolic extract of* C. membranaceus*.

## 2. Materials and Methods

### 2.1. Plant Extract

*C. membranaceus* roots were harvested from the forest area of the Eastern Region of Ghana to minimize the presence of contaminating herbicides and pesticides. The root product was carefully extracted with ethanol according to the protocol of Appiah [10]. Possible variation of content from batch to batch was averted by bulk harvest and fingerprinting. The extract was carefully checked for putative active ingredients(s) using chemical and biological parameters. Analyses for lack of contamination by pesticides, herbicides, heavy metals, microbes, and toxins were carried out as part of the quality control procedures. The Center for Plant Medicine Research (Mampong-Akuapem) further processed the freeze-dried extract into capsules (10 mg per capsule) under strict quality control procedures, with a recommended dose of 2 capsules t.i.d. The product has been registered with the Food and Drug Authority (FDA) Ghana as URO 500 after satisfying the FDA's requirement.

### 2.2. Experimental Site

Eleven (11) centers nationwide have been allowed by the Ministry of Health, Ghana, to serve well-tested and prepared medicinal plants products, alongside orthodox drugs. The Ghana Police Hospital, Accra, which was established in 1970 is a medium-size hospital with 100 beds. The hospital is currently undergoing expansion into an ultramodern 400-bed facility. It serves as one of the centers currently approved by the Ministry of Health to administer medicinal plant products alongside orthodox medicine. The center was therefore selected for this study.

### 2.3. Patients

The study obtained ethical clearance from the University of Ghana School of Biomedical and Allied Health Sciences, with ethics clearance number SAHS-ET/SAHS/PSM/ML/09/AA/26A/2012-2013 for an observational study to be conducted. The study complied with the Helsinki Declaration of 1964, with revision in October 2008. Patients opting for the use of the herbal remedy and who were willing to be part of the study were given informed consent forms. Forms were duly filled and the group formed the cohort. Patients were between the ages of 50 and 72 years. Furthermore, patients came from all walks of life; however, most of them were from the capital city of Ghana, Accra, with few from Togo and Nigeria. The cohort of 33 patients who opted for phytomedicine was not on any other medication for BPH management. Furthermore, these patients were under medical supervision of the urologist on the protocol development team. Patients were advised to carefully note any adverse effects and immediately report such, using the hotline provided.

### 2.4. Research Instruments

Four main research instruments were employed. The first was the International Prostate Symptom Score (IPSS) questionnaire followed by the second, the International Index of Erectile Function (IIEF) questionnaire. The third research instrument was blood sampling for routine and special biochemical tests and the last, abdominopelvic ultrasonography. Research instruments were employed before and after 3 months of treatment.

#### 2.4.1. IPSS Questionnaire

The IPSS is constructed on answers to seven questions concerning urinary symptoms and one question concerning QoL. Each question concerning symptoms in passing urine allowed the patient to choose 1 out of 6 answers representing increasing severity of a particular symptom. The answers were assigned points from 0 to 5, with 5 signifying worsening symptoms. The total score therefore ranged from 0 to 35 (asymptomatic to very symptomatic). Symptoms were graded alongside the following: incomplete emptying, frequency, intermittency, urgency, weak stream, straining and nocturia. These first 7 questions of the IPSS were identical to the questions appearing on the American Urological Association (AUA) Symptom Index. The eighth question dealt with the patient's perceived QoL and was scored 0–6, with 6 representing a terrible quality of life. Computed results were categorized as follows: mild (symptom score less than or equal to 7), moderate (symptom score range 8–19), and severe (symptom score range 20–35).

#### 2.4.2. IIEF Questionnaire

IIEF was based on the effects of erectile problems on one's reproductive health life over the past 4 weeks. The 15-point questionnaire finally categorized answers into broad areas of erectile function, orgasmic function, sexual desire, intercourse satisfaction, and overall satisfaction. Clinical interpretation ranged from the lowest score (no dysfunction) to the highest (severe dysfunction).

#### 2.4.3. Biochemical Assays

Five milliliters (5 mL) of blood samples was obtained from the patients before and after treatment for various blood tests as part of the urologist's request. Blood examinations were of two categories: routine examinations and special examinations.

*(1) Routine Examinations*. Renal function test (RFT), liver function test (LFT), and the lipid profile were performed. RFT was made up of urea, creatinine, Na^+^, and K^+^. LFT comprised total-direct and indirect bilirubin (TBIL, DBIL, and IND BIL, resp.), aspartate transaminase (AST), alanine transaminase (ALT), gamma-glutamyl transferase (GGT), alkaline phosphatase (ALP), total protein (TP), and albumin (ALB). Total cholesterol (TC), triglyceride (TG), high density lipoprotein cholesterol (HDL), low density lipoprotein cholesterol (LDL), and apolipoproteins A and B made up the lipid profile. LDL was calculated using Friedeweald's equation ([LDL-chol] = [Total chol] − [HDL-chol] − ([TG]/2.2)). All routine examinations were performed using the Vitros 5, IFS Chemistry analyzer (New York, USA). 

*(2) Special Examinations*. Free and total PSA (fPSA and tPSA, resp.) were performed using Accu-Bind free and total PSA ELISA kits were purchased from Monobind Inc. (California, USA). The tests were performed according to the manufacturer's instructions. In brief, serum samples were added alongside standards to a highly specific monoclonal anti-PSA antibody coated onto the surface of wells. After the first wash, an antibody-HRP enzyme conjugate was applied to form a sandwich complex on the well surface. Excess reagents were washed off followed by the addition of 3,3′,5,5′-tetramethylbenzidine (TMB)/hydrogen peroxide (substrate) to react with the HRP. After stopping the reaction with sulfuric acid, the final chromogen was then read at 450 nm using a microplate reader. PSA ratio was calculated as fPSA/TPSA in %.

#### 2.4.4. Abdominopelvic Scan

The SonoScape Digital Colour Doppler Ultrasound System 551–6000 (Shenzhen, China) was used. Patients were asked to drink approximately 1.5 L of water and allowed to wait for 1-2 hours before the scan was performed. The purpose of this preparation was to get the urinary bladder filled with urine so as to act as an acoustic window through which the prostate could better be visualized. Patients were then positioned on the ultrasound couch. With the pelvic area exposed, a liquid gel was applied on the probe surface to improve the contact between the patient's skin and the probe surface. The prostate volume and bladder volume at full capacity were obtained. After the scanning procedure, patients were then asked to void urine after which a second scan to determine the postvoid residual volume of the urinary bladder was performed.

### 2.5. Statistical Analysis

Data for the study was analyzed using Graphpad Prisms 6.01. Student's* t*-test for paired data was performed. Descriptive statistics was presented as mean ± SD. Pearson's correlation analysis was performed to determine associations.

## 3. Results

### 3.1. Demographic Data

In all, 30 clinically diagnosed BPH patients attending the urology and phytomedicine clinics with an average age of 66 ± 11 years completed the 3-month observational study. 3 others were excluded for noncompliance. No report of adverse effects was registered by any of the patients during the study. However, one diabetic patient reported improved glycemic index which further supports our unpublished data of possible hypoglycemic effect of* C. membranaceus*.

### 3.2. IPSS

IPSS relates to the degree of BPH or prostate cancer symptoms experienced. Only BPH patients were used for the study. Thirty-seven percent (37%) of the patients had severe prostate symptoms, 40% moderate symptoms, and 23% mild symptoms before treatment with* C. membranaceus* root extract. Fifty-seven (57%) and 43% of the patients had moderate and mild symptoms, respectively, after treatment. However, there was no patient with severe prostate symptom after treatment.

**Figure 1:**
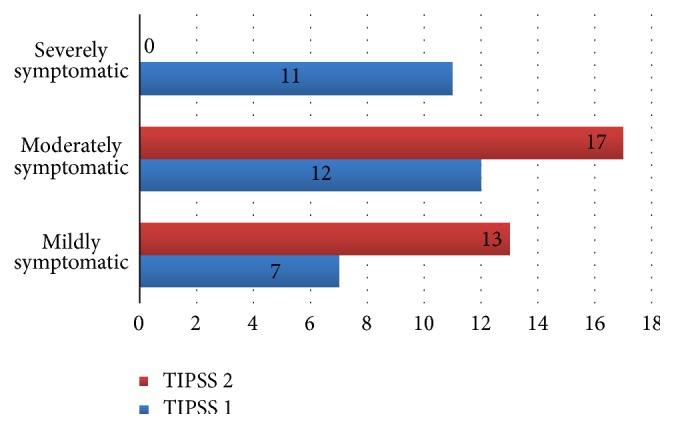
A diagram showing the prostate symptom score (IPSS) before and after 3 months of plant extract administration.

**Figure 2:**
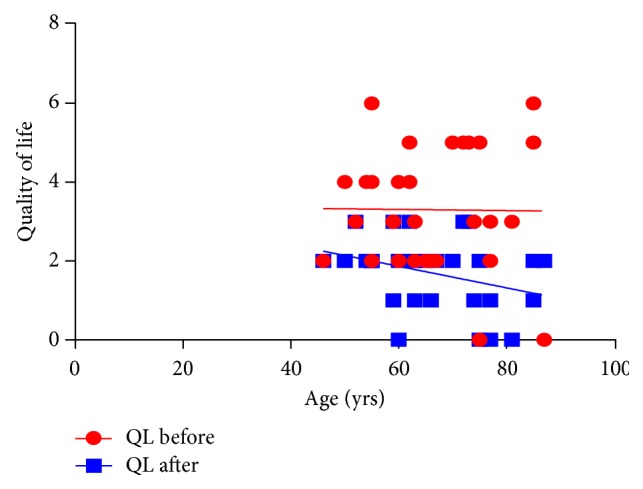
A relationship between age and QoL before (b) and after (a) treatment. *r* (b) = −0.010, *r* (a) = −0.330 and *P* value (b) = 0.956, *P* value (a) = 0.075.

**Figure 3:**
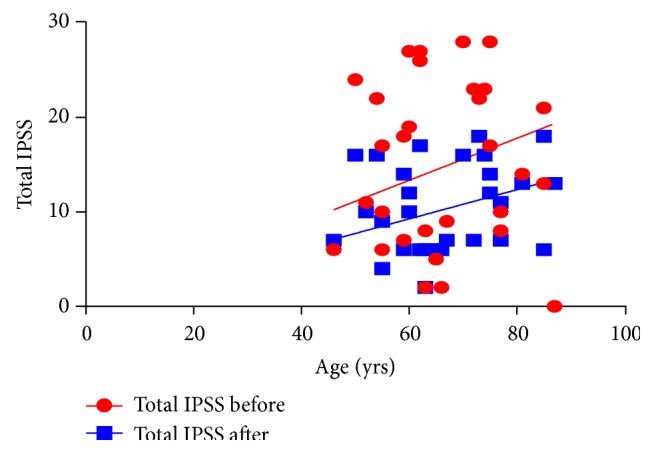
A relationship between age and IPSS before (b) and after (a) treatment. *r* (b) = 0.005, *r* (a) = 0.247 and *P* value (b) = 0.981, *P* value (a) = 0.189.

**Figure 4:**
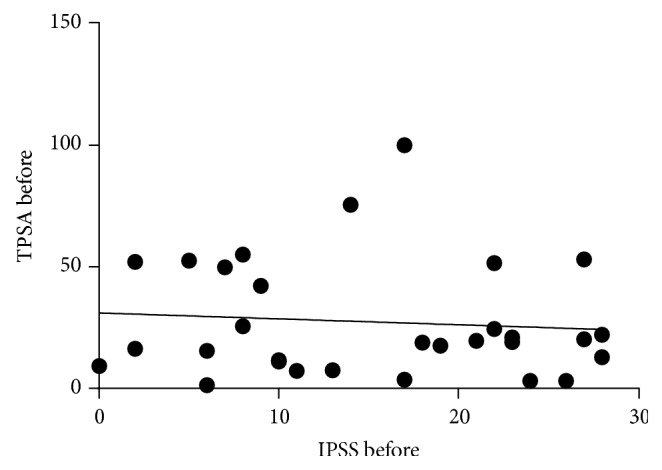
A correlation between tPSA and IPSS before treatment. *r* = −0.089, *P* value = 0.640.

**Figure 5:**
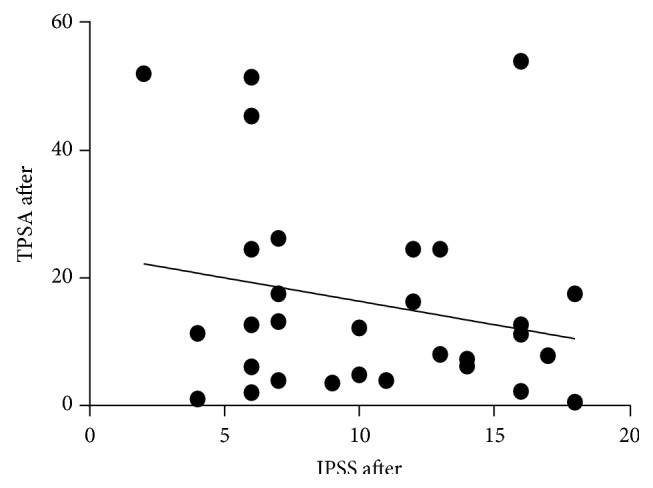
A correlation between tPSA and IPSS after treatment. *r* = −221, *P* value = 0.239.

IPSS improved significantly from 15.1 ± 8.7 units to 10.3 ± 4.7 units, signifying improved symptoms. The decrease was significant (*P* = 0.005). Additionally, QoL improved and this was highly significant (*P* = 0.001) (Table 1). Overall, prostate symptom score indicated that severe symptoms disappeared after treatment, while mild and moderate symptoms increased (Figure 1). In general, 33% of the patients recruited for this study were happy about their state of health before treatment while 67% of them were unhappy. After treatment, 83% were happy about their present state of health while 17% remained unhappy. A negative correlation between QoL and age was observed after treatment, although this was not significant (Figure 2). Furthermore, total IPSS did not significantly depend on age (Figure 3) or tPSA levels (Figures 4 and 5).

### 3.3. IIED

Thirty percent (30%) of the patients had severe erectile dysfunction, 40% moderate dysfunction, 24% mild-moderate dysfunction, 3% mild dysfunction, and 3% no dysfunction before treatment. After treatment, 20% of the patients had severe erectile dysfunction, 43% moderate, and 37% mild-moderate dysfunction. No patient had mild dysfunction or no dysfunction after treatment.

With regard to orgasmic dysfunction, 53% had severe orgasmic dysfunction, 27% moderate, 13% mild-moderate 4% mild and 3%, no dysfunction before treatment. After treatment, 37% had severe orgasmic dysfunction, 57% moderate, 3% mild-moderate, 0% mild and 3%, no dysfunction.

Patients with severe dysfunction in sexual desire formed 6%. Twenty percent (20%), 37%, 27%, and 10% had moderate, mild-moderate, mild, and no dysfunction in sexual desire, respectively, before treatment compared to 10% severe, 27% moderate, 50% mild-moderate 6% mild, and 7% no dysfunction, after treatment. Additionally, 40% of the patients had severe dysfunction, 33% moderate, 24% mild-moderate 3% mild, and 0% no dysfunction in intercourse satisfaction before treatment. After treatment, 23% had severe dysfunction, 67% moderate 7% mild-moderate, 3% mild dysfunction, and 0% no dysfunction.

**Figure 6:**
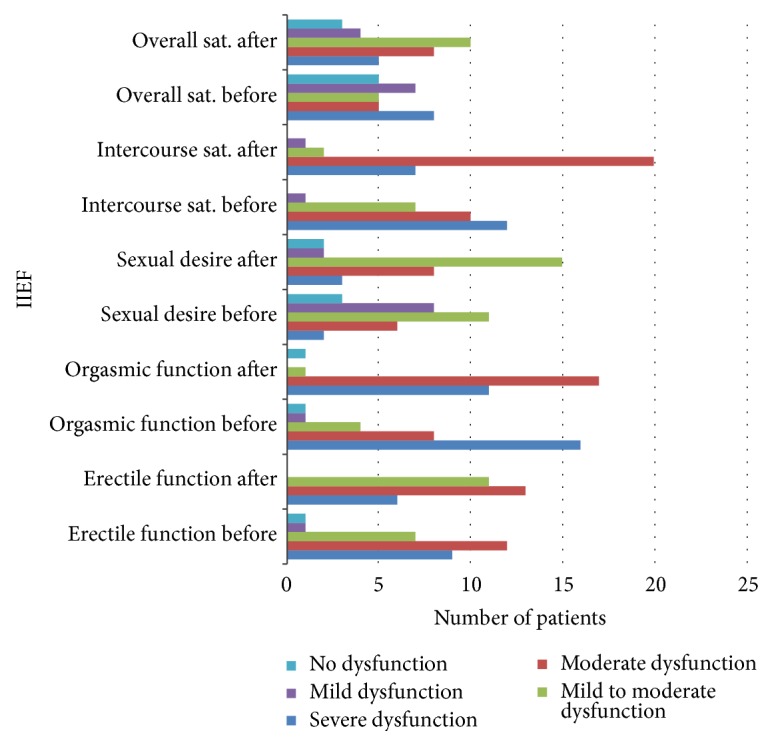
A diagram showing the general overview of patient's condition before and after treatment using the IIEF questionnaire.

**Figure 7:**
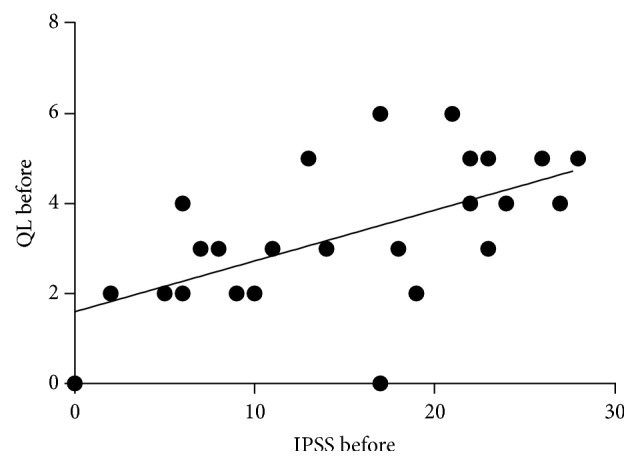
A correlation between QoL and IPSS before treatment. QoL before treatment strongly depended on IPSS. *r* = 0.634, *P* value = 0.002.

**Figure 8:**
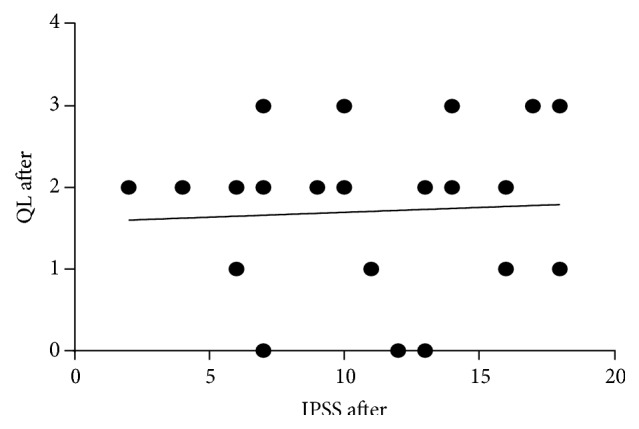
A correlation between QoL and IPSS after treatment. QoL appear to be stable and not dependent on IPSS. *r* = 0.061 and *P* = 0.747.

With the patient's assessment on overall sexual satisfaction, 27% had a severe dysfunction, whilst 17%, 17%, 23% and 16% of the rest of the patients had moderate, mild-moderate, mild and no dysfunction after treatment. Severe ED decreased with an increase in moderate symptoms (Figure 6). Furthermore, severe orgasmic dysfunction decreased after treatment with an increase in moderate dysfunction. With regard to sexual desire, mild to moderate dysfunction increased after treatment. Severe and mild to moderate intercourse satisfaction dysfunction decreased while moderate dysfunction increased. Severe dysfunction of overall satisfaction decreased, with an increase in mild and no dysfunction (Figure 6). Furthermore, there was a significant positive association between the QoL and IPSS before treatment (*P* = 0.002) (Figure 7). IPSS decreased after treatment with stable QoL (Figure 8).

**Figure 9:**
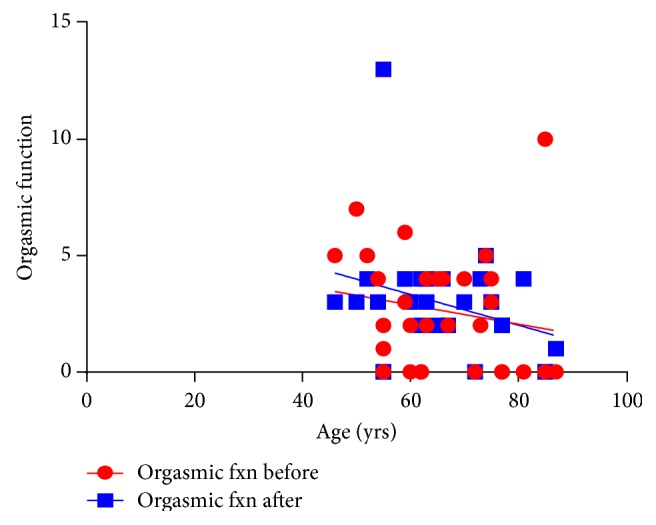
A relationship between age and orgasmic function before (b) and after (a) treatment. Orgasmic function remained relatively unchanged. Before *r* = −0.179, *P* = 0.344; after *r* = −0.303, *P* = 0.103.

**Figure 10:**
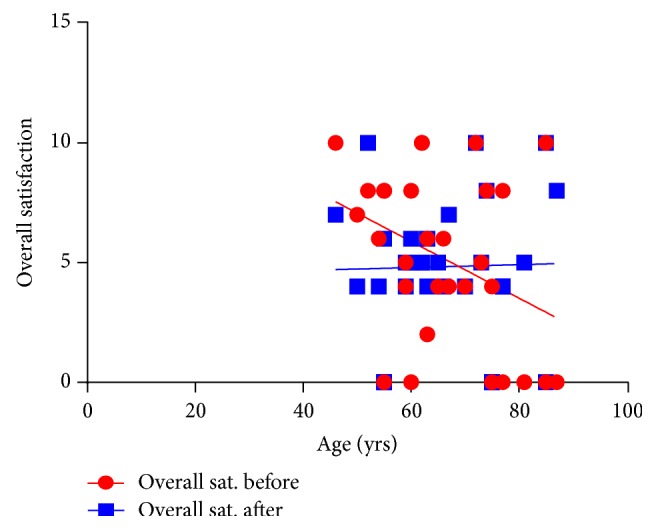
A relationship between age and the overall patient satisfaction before (b) and after (a) treatment. Overall satisfaction was determined by age before treatment. This no longer existed after treatment. Overall satisfaction appears to have stabilized. Before *r* = −0.364, *P* = 0.048 and after *r* = 0.024, *P* = 0.901.

In terms of age, as age increased overall satisfaction with “prostate health” decreased. This correlation however no longer existed after treatment (Figure 8). Correlation between ED and age indicated that ED was no longer age-dependent after treatment and had improved (Figure 9). Other insignificant associations with age occurred for sexual desire, intercourse satisfaction. However, overall satisfaction which was significantly age-dependent before treatment was no longer a factor after treatment (Figure 10).

### 3.4. Biochemical Results

#### 3.4.1. Routine Examinations

From Table 2, RFT remained relatively unchanged after the 3-month study. All LFT parameters increased slightly except for ALP that decreased slightly but insignificantly. However, significant increases were observed in TBIL and IND BIL (*P* = 0.001, *P* = 0.001, resp.) (Table 3). The lipid profile remained virtually unchanged except for APO A that increased significantly (*P* = 0.025) (Table 4).

#### 3.4.2. Special Examinations

From Table 5, total PSA (tPSA) reduced significantly from 27.9 ± 19.0 to 16.2 ± 11.8 ng/mL (*P* = 0.002). Furthermore, free PSA (fPSA) reduced from 6.1 ± 4.8 to 3.9 ± 2.9 ng/mL (*P* = 0.045). However, PSA ratio did not significantly change.

### 3.5. Abdominopelvic Scan

Data from the abdominopelvic scan showed that mean prostate volume reduced from 101.8 ± 41.3 to 54.5 ± 24.8 cm^3^. The change over the 3-month period was significant (*P* = 0.023).

## 4. Discussion

BPH continues to be a major problem worldwide. While new pharmaceutical drugs are being sought for, a gradual but substantive shift to nutraceuticals and medicinal plants is occurring globally. Medicinal plants are being used by 80% of the world's population. In emerging economies, it is the first line of medical support. Nonetheless in some advanced economies like Germany and Austria 90% use phytotherapeutic agents [7]. The drive towards the use of phytotherapeutic agents is a decision based on safety and side effects. Most medicinal plants have been used for ages and are considered safe with fewer side effects.

*C. membranaceus* aqueous root extract has been used in Ghana for decades for BPH treatment. The last decade has however witnessed a surge in scientific data on the effect of* C. membranaceus in vitro* and* in vivo* (animal studies).

Animal studies using normal Sprague-Dawley (S-D) rats demonstrated shrinking of the prostate and reduction in epithelial cells upon* C. membranaceus* administration [19]. Further to this, BPH models (S-D rats) also demonstrated prostate volume and prostatic index reduction in* C. membranaceus* treated rats alongside a finasteride positive control [16, 20]. Having obtained positive results in the aforementioned studies, it was incumbent to further demonstrate evidence of effectiveness to back anecdotal claims.

Improvement of urinary symptoms and QoL are important issues for decision making in the management of BPH. Clinical symptoms of BPH can be categorized into obstructive or irritative symptoms. The former is associated with the narrowing of the prostatic urethra with subsequent difficulty in passing urine or weak or trickling stream of urine, while the latter relates to sensation of incomplete bladder emptying, urgency, nocturia, and pollakiuria. Both categories affect the QoL. IPSS is a patient's assessment index used to rank severity of symptoms and QoL [21].

The IPSS questionnaire has been used extensively in many phytotherapeutic studies. Some polyherbal products have been documented to improve IPSS.* Chimaphila umbellata*,* Populus tremula*,* Pulsatilla pratensis*,* Equisetum arvense*,* Triticum aestivum* [22] and* Chimaphila umbellata* (L.) Barton,* Populus tremula*,* Pulsatilla pratensis* (L.) Mill.,* Equisetum arvense* L., and* Triticum aestivum* L [23] are made up of 5 polyherbal plants. Others like* Serenoa repens* (Bartram) Small/*Urtica dioica* L are biherbal preparations [24, 25]. Both the polyherbal and biherbal preparations have been documented to reduced IPSS. A few monoherbal plants have reduced IPSS. These include* Serenoa repens* Bartram [34],* Urtica dioica* L. [27], and* Pteris multifida* [28]. In the present study* C. membranaceus* ethanolic root extract (a monoherbal preparation) showed a significant reduction in total IPSS of the subjects at the end of the study period (*P* = 0.001). Furthermore, QoL improved significantly (*P* = 0.001). Admittedly, BPH symptoms impeach on QoL of subjects. This improvement was not age related (Figure 7). Additionally, a significant change in QoL was noticed. Although other factors in the IPSS questionnaire such as erectile function, sexual desire, and overall satisfaction improved, changes were not significant. However, overall satisfaction was significantly higher in younger men, and age differences did not exist after treatment. On the down side, orgasmic function and overall intercourse satisfaction were slightly worse (Table 1). Intercourse satisfaction is perhaps an age related perception as older men showed less satisfaction before treatment than younger men. However, some degree of improvement seen was largely due to the reduction in severe dysfunction related to intercourse (Figure 9). It appears that* C. membranaceus* primarily targets only the prostate without adverse effects on sexual function. This is of interest since 5-*α*-reductase inhibitors and *α*-1-adrenoceptor blockers affect erectile function. The improved QoL and IPSS may largely therefore be related to relief of symptoms. It is worthy to note that sexual desire after treatment was almost significant. This will need further investigations to determine whether* C. membranaceus* possess some aphrodisiac properties.

Severe ED, however, dropped after treatment (Figure 6). Further support to this is the fact that severe lower urinary tract symptoms (LUTS) disappeared after 3 months with a corresponding increase in those with mild and moderate symptoms (Figure 6). The improvement could be attributed to improved urination although such directly related parameters were not measured.* Vaccinium macrocarpon* Aiton is an example of a monoherbal plant that demonstrated improved LUTS after administration of its extract [29]. Further evidence of improved IPSS is accompanied by reduced PSA levels. Most medicinal plant studies (in animals and humans) did not measure PSA after the treatment period. However, Engelhardt and Riedl reported a significant decrease in IPSS and PSA after one-year isoflavone extract from red clover [30]. This study also reports not only improved IPSS but also significant reduction in both tPSA and fPSA (Table 2).

Total PSA is the sum of both bound and free PSA; however, free PSA is assessed only if the total PSA is increased. PSA is primarily a tissue-specific marker. From an elevated PSA measurement, it is difficult to distinguish between a benign and malignant transformation of the prostate gland. Differentiating between the two is where free PSA is useful. Free PSA is more often formed from benign transformations while bound PSA tends to come from malign transformations [31]. In this study both fPSA and tPSA were found to be significant. However, the ratio was not significant before and after treatment. Collectively, the free : total PSA ratio can be used as an additional marker for prognosis of hormone treatment [32].

Total and indirect (unconjugated) bilirubin levels significantly increased in this study. However levels were within the normal reference interval. Such physiological increases are beneficial and bilirubin has been demonstrated to be an effective antioxidant using isolated heart mitochondria [33].

Apolipoprotein A-I is the major protein component of high density lipoprotein (HDL) in plasma. Chylomicrons secreted from the intestinal enterocyte also contain apo A-I, but it is quickly transferred to HDL in the bloodstream [34]. The protein promotes fat efflux, including cholesterol, from tissues to the liver for excretion. Apolipoprotein A-I itself also removes seeding molecules for oxidation from the arterial wall and also facilitates reverse cholesterol transport. In this study Apo A was significantly elevated after treatment in tandem with an increase in HDL which was almost significant. Hence* C. membranaceus* appears to have antiatherogenic properties as observed in preclinical studies [35].

Further clinical significance is the reduction in prostate volume. Indeed two hypotheses exist concerning the pathoetiology of BPH. Prostate enlargement has been attributed to the accumulation of dihydrotestosterone (DHT) (which may cause cellular hyperplasia) and an increase in prostatic estrogen levels with age. Reduced prostate volume and prostatic index have previously been shown with the use of* C. membranaceus* aqueous root extract in animal models [17, 19, 35].* Secale cereale* in combination with* Serenoa repens* is said to reduce prostate volume in animals [36]. Some studies using* Cucurbita pepo* L. and* Lepidium meyenii* as monoherbal preparations documented reduced prostate volume in animal models [37, 38]. Only few human studies with phytomedicines have demonstrated prostate volume reduction. In a meta-analysis by Azimi et al. [39], 32 human studies involving the use of medicinal plants were documented. PR-2000, a polyherbal plant extract (made up of* Tribulus terrestris* L.,* Caesalpinia bonducella* (L.) Fleming,* Asparagus racemosus* Willd.,* Areca catechu* L., and* Crataeva nurvala* Buch-Ham) reduced prostate volume after 6 months treatment [21]. Two monoherbal plants were said to have shrank the prostate. Of the two, the study of Safarinejad [40] reported mild shrinkage using* Urtica dioica* L. extract. The other by Engelhardt and Riedl [30] used 60 mg/day isoflavone from* Trifolium pretense* (red clover) on 20 BPH patients and reported a reduction in prostate volume and PSA and improved IPSS. In that study PSA reduced significantly by 33% from 10.16 ng/mL to 7.15 ng/mL. In this study the PSA reduced significantly by 40.8% from 27.4 ng/mL to 16.2 ng/mL. Reduction of PSA observed in this study corroborates findings from preclinical studies [17], and this could be attributed to its possible 5*α*-reductase inhibitory properties. Prostate volume shrinkage after 12-month treatment in the study of Debruyne et al. [26] was modest and insignificant (49.3 cm^3^ to 44.3 cm^3^). Of more serious consequences in that study, is the fact that all three liver transaminases significantly increased (*P* < 0.001) at the end of the study, signifying commencement of liver damage. In this study, prostate volume reduced significantly by 46.6% after 3-month* C. membranaceus* administration (*P* = 0.023). Liver function, renal function tests, and lipid profile remained normal.

## 5. Conclusion

In conclusion, the ethanolic root extract of* C. membranaceus* is one of the few monoherbal products that remarkably reduces PSA levels, prostate volume, and subsequently improves the QoL of patients with BPH. There is the need for a larger multicentered clinical trial to further confirm its efficacy and beneficial effects.

## Figures and Tables

**Table 1:** Table showing prostate symptom score, sexual function, and quality of life in BPH patients after three (3) months of *C.  membranaceus* ethanolic root extract administration at 20 mg t.i.d.

Parameter	Mean ± SD (before)	Mean ± SD (after)	*P* value
Age (yrs)	66.0 ± 11.0		
Total IPSS	15.1 ± 8.7	10.3 ± 4.7	0.001^∗^
Erectile function	10.0 ± 6.0	9.7 ± 4.1	0.751
Orgasmic function	2.6 ± 2.5	2.9 ± 2.4	0.620
Sexual desire	5.9 ± 2.5	5.0 ± 2.1	0.053
Intercourse satisfaction	3.8 ± 3.2	4.6 ± 2.5	0.154
Overall satisfaction	5.2 ± 3.6	4.8 ± 2.9	0.598
Quality of life	3.3 ± 1.6	1.7 ± 0.9	0.001^∗^

^∗^Significance at 95% confidence interval.

**Table 2:** A table showing renal function test (RFT) before and three (3) months after administration of *C.  membranaceus* ethanolic root extract at 20 mg t.i.d.

Parameter	Mean ± SD (before)	Mean ± SD (after)	*P* value
Sodium (mmol/L)	142.8 ± 6.6	142.2 ± 6.6	0.672
Potassium (mmol/L)	4.3 ± 0.6	4.3 ± 0.6	0.609
Urea (mmol/L)	4.3 ± 1.4	4.4 ± 1.3	0.664
Creatinine (*μ*mol/L)	105.9 ± 26.5	106.0 ± 26.2	0.981

**Table 3:** A table showing liver function test (LFT) before and three (3) months after *C*.*  membranaceus* ethanolic root extract administration at 20 mg t.i.d.

Parameter	Mean ± SD (before)	Mean ± SD (after)	*P* value
AST (U/L)	17.8 ± 4.4	18.6 ± 5.1	0.391
ALT (U/L)	16.9 ± 6.5	17.9 ± 5.7	0.465
GGT (U/L)	34.9 ± 15.3	35.8 ± 19.2	0.729
ALP (U/L)	76.6 ± 52.3	68.8 ± 20.6	0.441
TP (g/L)	74.4 ± 6.9	75.3 ± 9.0	0.495
ALB (g/L)	44.2 ± 4.8	46.0 ± 7.2	0.092
TBIL (*μ*mol/L)	4.9 ± 2.6	8.3 ± 4.7	0.001^∗^
DBIL (*μ*mol/L)	2.9 ± 2.2	3.4 ± 2.5	0.347
IND BIL (*μ*mol/L)	2.0 ± 1.8	4.8 ± 4.5	0.001^∗^

^∗^Significance at 95% confidence interval.

**Table 4:** A table showing serum lipid levels before and three (3) months after *C.  membranaceus* ethanolic root extract administration at 20 mg t.i.d.

Parameter	Mean ± SD (before)	Mean ± SD (after)	*P* value
TC (mmol/L)	5.03 ± 1.12	5.19 ± 1.15	0.423
TG (mmol/L)	1.15 ± 0.43	1.22 ± 0.56	0.518
HDL (mmol/L)	0.76 ± 0.33	0.88 ± 0.32	0.063
LDL (mmol/L)	3.74 ± 1.03	3.75 ± 1.02	0.959
APO A (g/L)	1.31 ± 0.45	1.51 ± 0.47	0.025^∗^
APO B (g/L)	0.58 ± 0.37	0.49 ± 0.13	0.222
APO B/A ratio	2.81 ± 1.47	3.24 ± 1.27	0.067

^∗^Significance at 95% confidence interval.

**Table 5:** Table showing the different levels of total PSA (tPSA), free PSA (fPSA), PSA ratio, and prostate volume before and after three (3) months of administration of *C.  membranaceus* ethanolic root extract at 20 mg t.i.d.

Parameter	Mean ± SD (before)	Mean ± SD (after)	*P* value
Total PSA (ng/mL)	27.4 ± 19.0	16.2 ± 11.8	0.002^∗^
Free PSA (ng/mL)	6.1 ± 4.8	3.9 ± 2.9	0.045^∗^
PSA ratio (%)	23.9 ± 18.7	24.6 ± 16.4	0.813
Prostate volume (cm^3^)	101.8 ± 41.3	54.5 ± 24.8	0.023^∗^

^∗^Significance at 95% confidence interval.
