# Erratum to “Shrinkage of Prostate and Improved Quality of Life: Management of BPH Patients with* Croton membranaceus* Ethanolic Root Extract”

**DOI:** 10.1155/2019/5958036

**Published:** 2019-02-05

**Authors:** Evidence-Based Complementary and Alternative Medicine

In the article titled “Shrinkage of Prostate and Improved Quality of Life: Management of BPH Patients with* Croton membranaceus* Ethanolic Root Extract” [1], an administrative error meant that the editor originally named on the article did not handle the review process. The article and its peer review have been reassessed by Giuseppe Morgia, who found no concerns.
